# Mutational Analyses of the Cysteine-Rich Domain of Yvh1, a Protein Required for Translational Competency in Yeast

**DOI:** 10.3390/biology11081246

**Published:** 2022-08-22

**Authors:** Hannah Zang, Robert Shackelford, Alice Bewley, Alexander E. Beeser

**Affiliations:** 1 Duke University School of Medicine, Durham, NC 27708, USA; 2 Logan University, St. Louis, MO 63017, USA; 3 Washington University School of Medicine, St. Louis, MO 63110, USA; 4 Lyon College Division of Natural Sciences, Batesville, AR 72501, USA

**Keywords:** Yvh1, Mrt4, ribosome maturation, nascent protein translation

## Abstract

**Simple Summary:**

Ribosome maturation in eukaryotes is a highly energy-consuming, evolutionarily conserved process, directed by a large number of auxiliary proteins (>200) called *trans*-acting factors (TAFs). TAFs direct the ordered and directional assembly of immature pre-40S and pre-60S complexes in multiple cellular compartments, but they are not themselves components of translationally competent ribosomes. Although the enzymatic properties of many TAFs are understood, others assist ribosome maturation without predicted enzymatic activity. An important late-acting 60S TAF is Mrt4, which initially binds to pre-60S complexes within the nucleus that must be released from pre-60S complexes following translocation into in the cytoplasm prior to the addition of a complex of acidic phosphoproteins denoting the ribosomal stalk, an essential component of the peptidyl transferase center (PTC) of ribosomes. Prior genetic studies implicated the dual-specificity phosphatase Yvh1 as the protein responsible for cytoplasmic Mrt4 removal, but how Yvh1 facilitates this is unknown. *yvh1*Δ strains are viable but grow slowly due to translational defects associated with improper ribosome assembly. Yvh1-dependent Mrt4 removal is independent of YVH1’s protein phosphatase domain but instead maps to an evolutionarily conserved cysteine-rich domain (CRD) of unknown function. In order to better understand how YVH1’s CRD facilitates Mrt4 removal and thus 60S maturation, we identified loss-of-function (LOF) variants of Yvh1 incapable of displacing Mrt4 precluding the addition of the ribosomal stalk. This approach additionally identified a variant of Yvh1 (Yvh1F283L) that functions as an expression-dependent, dominant-negative variant capable of perturbing ribosome assembly in cells containing wild-type YVH1. These findings are consistent with, and expand on, recent structural models for Yvh1-dependent Mrt4 removal from pre-60S complexes and generate novel first-generation probes that can be used to better understand eukaryotic ribosomal maturation.

**Abstract:**

Ribosome assembly is a complex biological process facilitated by >200 *trans*-acting factors (TAFs) that function as scaffolds, place-holders or complex remodelers to promote efficient and directional ribosomal subunit assembly but are not themselves part of functional ribosomes. One such yeast TAF is encoded by Mrt4 which assembles onto pre-60S complexes in the nuclear compartment and remains bound to pre-60S complexes as they are exported into the cytoplasm. There, Mrt4 is displaced from pre-60S complexes facilitating the subsequent addition of the ribosomal stalk complex (P0/P1/P2). Ribosomal stalk proteins interact with translational GTPases (trGTPase) which facilitate and control protein synthesis on the ribosome. The rRNA-binding domain of Mrt4 is structurally similar to P0, with both proteins binding to the same interface of pre-60S subunits in a mutually exclusive manner; the addition of the ribosomal stalk therefore requires the displacement of Mrt4 from pre-60S subunits. Mrt4 removal requires the C-terminal cysteine-rich domain (CRD) of the dual-specificity phosphatase Yvh1. Unlike many other TAFs, yeast lacking Yvh1 are viable but retain Mrt4 on cytoplasmic pre-60S complexes precluding ribosomal stalk addition. Although Yvh1’s role in Mrt4 removal is well established, how Yvh1 accomplishes this is largely unknown. Here, we report an unbiased genetic screen to isolate Yvh1 variants that fail to displace Mrt4 from pre-60S ribosomes. Bioorthogonal non-canonical amino acid tagging (BONCAT) approaches demonstrate that these YVH1 loss-of-function variants also display defects in nascent protein production. The further characterization of one LOF variant, Yvh1F283L, establishes it as an expression-dependent, dominant-negative variant capable of interfering with endogenous Yvh1 function, and we describe how this Yvh1 variant can be used as a novel probe to better understand ribosome maturation and potentially ribosome heterogeneity in eukaryotes.

## 1. Introduction

Ribosomes are very large ribonucleoprotein (RNP) complexes responsible for translating genomic information into proteins in all living cells. Eukaryotic ribosomes are exceedingly complex machines consisting of a large (60S) and small (40S) ribosomal subunit that assemble with mRNA and other translation initiation factors to form a translationally competent ribosome (reviewed in [1]). The smaller 40S ribosomal subunit contains a single rRNA and 33 ribosomal proteins (RPs), whereas the larger 60S subunit contains 3 rRNAs and 46 RPs. Based on the centrality of ribosomal function, it is not surprising that ribosome biogenesis is a highly regulated process. Eukaryotic ribosome biogenesis is an evolutionarily conserved process that occurs in at least three distinct subcellular environments. In yeast, where this pathway is best characterized, ribosome assembly begins with RP translation. RPs are then imported into nuclear/nucleolar compartments where they associate with ribosomal RNAs (rRNAs) undergoing co- and post-transcriptional processing and other RPs to generate increasingly complex ribonucleoprotein complexes [2]. The directional maturation of these RNP complexes is not spontaneous and requires more than 200 additional proteins (denoted as *trans*-acting factors, TAFs) that facilitate the directionally appropriate pre-40S/60S maturation [3], but they are not themselves components of translationally competent ribosomes. Once appropriately assembled in the nuclear compartment, the still translationally incompetent pre-40S/60S complexes are then exported back into the cytoplasm, where the final maturation of both the large and the small subunit occurs. Afterwards, the 43S pre-initiation complex (PIC) associates with mRNA to form the 48S initiation complex which scans along the mRNA, stopping at the start codon. At this point, eukaryotic initiation factors (eIFs) are displaced, allowing for the addition of the 60S large ribosomal subunit and translationally competent 80S ribosomes [4,5,6].

The identities of many eukaryotic TAFs were initially identified from genetic screens in yeast. More recently, ribosomal complex assembly intermediates isolated from yeast strains harboring tandem affinity tagged ribosomal proteins have been elucidated via cryo-electron microscopy [7,8,9]. These approaches have been particularly informative in elucidating the dynamic changes in RNA conformation and protein exchanges, allowing for the addition of the ribosomal stalk that interacts with translational GTPases (trGTPases, reviewed in [10]) that control and facilitate protein synthesis. The ribosomal stalk contributes directly to the formation of the ribosomal peptidyl transferase center (PTC) [11] responsible for the two enzymatic activities of ribosomes: peptide bond formation and peptide release (reviewed in [1]). Central to 60S cytoplasmic maturation is the Mrt4 TAF. Mrt4 is an RNA-binding protein that associates with nuclear/nucleolar pre-60S subunits by forming specific contacts with 25S rRNA. Mrt4-containing pre-60S complexes assembled in the nucleus are transported from the nuclear compartment into the cytoplasm, where Mrt4 is displaced from pre-60S subunits and replaced with the P0 protein that forms the base of the ribosomal stalk. Mrt4 shares significant homology with the P0 *n*-terminal rRNA-binding domain, leading to the suggestion that the binding of Mrt4 or P0 is mutually exclusive [12] on pre-60S complexes. Importantly, Mrt4 lacks the C-terminal extension of P0 that allows for the recruitment the other acidic proteins (Rpp1a, Rpp1b, Rpp2a and Rpp2b) that interact with translation elongation factors [13]. This has led to a model whereby pre-60s complexes initially associate with Mrt4 within the nuclear compartment, and these Mrt4 containing pre-60S complexes are exported into the cytoplasm, where Mrt4 is displaced from the pre-60S subunits, allowing for the subsequent binding of P0 and other proteins that comprise the ribosomal stalk. Cells that cannot promote the exchange of Mtr4 for P0 fail to add to the ribosomal stalk and as a consequence exhibit defects in protein synthesis [4,5].

As Mrt4 removal obligatorily precedes the addition of the ribosomal stalk, it puts significant importance on the identification of the factors responsible for Mrt4 displacement from immature pre-60S ribosomes. In a series of papers, two labs independently identified the dual-specificity phosphatase, Yvh1, as the factor responsible for cytoplasmic Mrt4 removal [4,5]. Yvh1 is an atypical member of the dual-specificity phosphatase family, and yeast strains lacking Yvh1 are viable but exhibit a variety of pleotropic phenotypes [14,15,16,17,18] including, importantly, slow growth. As TAFs are enriched in proteins that promote RNP remodeling (snoRNPs, nucleases, helicases, ATPases and GTPases), significant efforts were invested to identify the Yvh1 substrate. These efforts led to a quite surprising result; the ability of Yvh1 to promote Mrt4 removal from pre-60S complexes was independent of both protein phosphatase activity and of the entire protein phosphatase domain [14] but mapped to an evolutionarily conserved cysteine-rich domain (CRD) that binds zinc at the protein’s C-terminus. Attempts to further characterize the role of the CRD in Mrt4 removal by targeting these evolutionarily conserved residues were largely unsuccessful due to issues of protein folding/stability in vivo and in vitro [19].

Here, in order to better understand how Yvh1 displaces Mrt4, we employed an unbiased genetic screen to identify novel loss-of-function (LOF) point mutations within the Yvh1 CRD that preclude Mrt4 displacement from maturing pre-60 ribosomal subunits. These LOF variants exhibited persistent cytoplasmic retention of Mrt4-GFP, preventing the addition of P0 and the ribosomal stalk proteins. As expected of strains unable to appropriately complete 60S maturation, the LOF variants predictably displayed a pronounced slow-growth phenotype and further demonstrated reduced nascent protein production compared to cells harboring wild-type YVH1. A particular LOF variant isolated from our genetic screen (Yvh1F283L) functioned as an expression-dependent, dominant-negative allele capable of perturbing nascent protein expression and growth in cells containing wild-type Yvh1. Collectively, we generated a panel of Yvh1 loss-of-function variants and demonstrated that one variant that may serve as a novel probe that can be used to genetically investigate the biological consequences of preventing a late step in ribosome biogenesis.

## 2. Materials and Method

### 2.1. Yeast Strains and Growth Conditions

All strains used were diploid and derived from the wild-type GYC86 (*Mata his3 leu2 ura3/ Matα his3 leu2 ura3*). GYC86 and the isogenic YVH1-deficient stain HPY120 (*Mata his3 leu2 ura3 yvh1::HIS3/Matα his3 leu2 ura3 yvh1::HIS3*) have been described previously [20,21]. These diploid strains were chosen as they are extensively characterized, demonstrate very robust and predictable galactose induction and, as diploids, they are larger than haploid strains, which facilitates microscopy. A derivative of HPY120 expressing the C-terminal tagged Mrt4 construct was made essentially similarly to the method described in [5]. The C-terminal Mrt4-GFP targeting construct was made with oligosAB00155 (5′-GCATAGCTCCACTGTTGAAAGCACTAACATCAACATGGAACGGATCCCCGGGTTAATTAA-3′) and AB00156 (5′-TGGTATATTAAAAAAGGCTTGCAACTCCTCGTTCAGCTTAGAATTCGAGCTCGTTTAAAC-3′) (IDT) and the parental vector pFA6a-GFP(S65T)KanMX as a template. pFA6a-GFP(S65T)-kanMX6 was a gift from Jurg Bahler and John Pringle (Addgene plasmid # 39292; https://www.addgene.org/39292/) (accessed 25 May 2022). The resulting purified PCR product was used the transform HPY120 to G418 resistance using LioAC/PEG. The correct integration of the GFP-KanMx cassette in frame with the C-terminal of Mrt4 was confirmed via polymerase chain reaction, with oligos annealing to the MRT4 open reading frame and 3′ UTR. Standard yeast growth media were used. Synthetic complete (SC) media consisted of 1.7 g of yeast nitrogen base (YNB), 5.0 g of ammonium sulfate, 20 g of carbon source (dextrose, raffinose or galactose) and --URA or --URA MET dropout powder, as recommended by the manufacturer (Sunrise Science Products), per liter. YEPD was 20 g of peptone, 20 g of dextrose and 10 g of yeast extract per liter. Plates contained 2% agar. For experiments with galactose induction assays, yeast transformants were grown overnight in liquid cultures at 30 °C and 250 rpm in raffinose –URA media. The next day, the cultures were diluted in the same media prewarmed to 30 °C and grown to an A600 of approximately 0.700 on a shaking incubator (250 rpm) before the addition of concentrated sterile galactose to 4% for the time indicated.

### 2.2. Plasmids

The parental Gal1,10 GST expression vector (pEGKG) and pAB36 (Gal1,10 GST-YVH1) have been previously described [14,21]. Plasmid pGST-Dusp12, encoding the human ortholog of Yvh1, DUSP12, was generated as follows: The human DUSP12 cDNA sequence was obtained from the Mammalian Gene Collection (MGC) clone 3958403. DUSP12 was amplified with oligos (5′-GCCCGGATCCATGTTGGAGGCTCCG-3′) and (5′-GCGAATTCTCATATTTTTCCTGT-3′) (IDT) using Pfusion polymerase (NEB, Ipswich, MA, USA). The resulting PCR product was digested with BamHI and EcoRI and cloned into the baculoviral expression vector pFBHTB (Invitrogen) and similarly digested. The resulting DUSP12 insert was sequenced completely, and then, the plasmid digested with BamHI and HindIII and the DUSP12 cDNA was ligated into pEGKG and similarly digested. The ectopic expression of DUSP12 is known to fully complement the phenotypes of *yvh1*Δ strains [5,19].

### 2.3. Construction of A Gapped Yvh1Δ123–364 Plasmid Library

Plasmid pAB36 (pGST-YVH1) was first linearized with *BglII* (NEB), which cut 367 nucleotides into the YVH1 open reading frame (corresponding to amino acid R123). The linearized plasmid was then digested with *SpeI* (NEB), which cut at position1012 (corresponding to amino acid 338 of Yvh1) and 1254 within the Yvh1 3′ UTR. This construct removed all 7 evolutionarily conserved cysteines between yeast and humans and the variant stated 5 amino acids C-terminal of the catalytic cysteine (C117) and extended 162 bp into the YVH1 3′ UTR. The linearized gapped plasmid lacked amino acids 123–364 of the YVH1 ORF. The gapped plasmid backbone was isolated from 1% TAE agarose gel with the Wizard^®^ SV Gel (Promega, Madison, WI, USA) and PCR Clean-Up System (Promega, Madison, WI, USA) and treated with Antarctic phosphatase (NEB), as recommended by the manufacturer, to reduce backbone recircularization. Plasmid pAB36 (pGST-YVH1) was used as a template with the following oligonucleotides (IDT): ABLyon1 (5′- GAAACGAACCGATTCATTGATCAATGC-3′)/ABLyon2 (5′-TGAAGATGACGTGGGAAGAGAGCATGA-3′). An error-prone polymerase chain reaction (EPPCR) was accomplished with *Taq* polymerase (NEB) in 1× reaction buffer containing variable amounts of MnCl_2_. The optimal MnCl_2_ concentration (200 mM) was empirically determined by subcloning EPPCR products into a TA vector (pXT cut with BstX1, a gift of Jonathan Chernoff, Fox Chase Cancer Center, Philadelphia, PA, USA) using blunt/TA ligase master mix (NEB). The transformation of the ligation reactions into *E. coli* JM109 was made competent by the Mix & Go! transformation kit (Zymo research, Tustin, CA, USA), and the substances were plated onto LB-Amp plates. Individual ampicillin-resistant EPPCR colonies were grown in LB-Amp media, and plasmids were isolated with the PureYield plasmid miniprep kit (Promega) and subjected to Sanger sequencing (Genewiz, Cambridge, MA, USA) to empirically determine MnCl_2_ concentrations that led to optimized but not excessive levels of mutagenesis.

### 2.4. GAP Repair by Homologous Recombination

Diploid strain HYP120 (*yvh1*::HIS3/*yvh1*::HIS3) was subjected to standard lithium acetate/PEG transformation with the parental vector (pEGKG, GST alone), the complementing positive control (pAB36, pGST-YVH1) or varying ratios of the gapped dephosphorylated pAB36 plasmid and the EPPCR fragments essentially as described in [22]. Yeast transformants were selected by plating on SD-URA plates and being grown at 30 °C. Smaller, and thus slower-growing loss-of-function (LOF) colonies were isolated and confirmed to be slow growing via repeated streaking onto SD -URA plates and via serial dilution spot assays on SD-URA plates.

### 2.5. Enrichment for Full-Length or near Full-Length LOF Clones

Candidate LOF transformants were isolated, grown in SD Raffinose -URA media to an A600 between 0.5 and 1.0. Sterile Galactose (40%) was added to a final concentration of 4% for 4–6 h to induce the expression of the GST-YVH1 variants. The cells were collected via brief centrifugation (3000 rpm for 5 min in IEC Centra GP8R), the supernatants were discarded and the pellet was resuspended in 1× Sample Buffer containing 2-mercaptoethanol (LI-COR, Lincoln Nebraska). The samples were boiled for at least 5 min and then frozen at −20 °C.

### 2.6. Western Blot

Yeast whole-cell lysates were loaded onto 10% SDS-PAGE gel and electrophoresed at 125–150 V in an NOVEX mini-gel chamber until the loading dye front ran off of the gel. Proteins were then electro-transferred to nitrocellulose (LI-COR) in transfer buffer (25 mM Tris, 192 mM glycine and 20% *v*/*v* Methanol) as recommended (LI-COR). Total protein was visualized by staining the nitrocellulose membrane with Revert protein Dye (LI-COR) and imaged at CW700 on a LI-COR Odyssey. The membranes were then blocked for at least one hour in 1× PBST + 4% non-fat milk. The blocked membrane was added to a seal-a-meal bag, and the anti-GST primary antibodies B-14 (1:5000) or A6 (1:2500) were added (Santa Cruz Biotechnology). Primary antibodies were incubated with rocking at 4 °C for at least one hour. The membrane was washed with excess 1× PBST 3 times for 10 min each, followed by the incubation of a CW800 goat anti-mouse secondary antibody (1:10,000) (LI-COR). Secondary incubation was for at least an hour at room temperature. The blot was washed 3X 5 min in 1× PBST before being imaged on the CW700 and CW800 channels of a LI-COR Odyssey to facilitate overlays.

For experiments where equivalent amounts of total protein were required, yeast cells were pelleted via brief centrifugation (3000 rpm for 5 min in IEC Centra GP8R). The supernatant was discarded, and the cell pellet was washed 2× with ice-cold sterile PBS. The resulting pellet was frozen overnight at −80 °C. The cell pellets were thawed on ice and resuspended in 1× PBST containing 1× Halt Protease inhibitor (ThermoFisher) then transferred to screw cap tubes containing 200 µL of 1.0 mm-diameter glass beads (BioSpec). Yeast was disrupted by 6 × 30 s pulses at maximum speed in a FastPrep FP120 (Thermo), where tubes were returned to ice between pulses to prevent warming. The screw cap tubes were centrifuged at 16,100× *g* at 4 °C in an Eppendorf 5150 R centrifuge for 10 min. The whole-cell lysate supernatants were transferred to sterile microfuge tubes, and protein concentrations were determined with the Pierce BCA Protein assay kit (ThermoFisher). Protein concentrations were equilibrated to the lowest concentration by dilution with the appropriate volume of lysis buffer (1× PBST with 1× HALT protease inhibitor).

### 2.7. Rescue of Candidate LOF Clones

Individual candidate LOF plasmids were rescued from yeast as described [23] with minor modifications. Briefly, 5 mls overnight cultures of candidate LOF transformants were grown in SC -URA media; then, the cells were collected via centrifugation. The cell pellet was washed via resuspension in 1.0 mL of sterile water, and the culture was then transferred to a 2.0 mL screw cap microfuge tube. The cells were centrifuged at 16,100 rpm for 60 s, and the supernatant was discarded. The cell pellet was resuspended in 100 µL of STET buffer (8% sucrose, 50 mM Tris pH 8, 50 mM EDTA and 5% Triton X-100). Approximately 0.25 g of 0.3 mm acid washed beads were added to the tube followed by vortexing at room temperature for 5 min at full speed. After vortexing, an additional 100 µL of STET buffer was added, and the tube was vortexed briefly and then placed in a boiling water bath for 3 min before placing the tube on ice. The tube was spun at full speed in a microfuge for 10 min at 4 °C, and 100 µL of the supernatant was carefully removed and transferred to a clean microfuge tube. To this supernatant, 50 µL of 7.5 M ammonium acetate was added, the tubes were mixed via vortexing followed by incubation for at least one hour at −20 °C. The microfuge tubes were then centrifuged at 16,100 rpm for 15 min at 4 °C. Then, 100 µL of the supernatant was removed and transferred to a clean tube containing 200 µL of ice-cold 95% ethanol; the tube was vortexed and then centrifuged again at 16,100 rpm at 4 °C for 10 min. The supernatant was carefully withdrawn and discarded, and the pellet was washed in −20 °C 70% ethanol. After brief centrifugation, the 70% ethanol was withdrawn and discarded, and the tubes were allowed to dry (open top) in a chemical fume hood for at least 15 min at room temperature. The pellets (containing the pGST-YVH1 candidate LOF plasmids) were resuspended in 20 µL of nuclease free water, and 10 µL was used to transform JM109 cells rendered competent with the Mix and Go! *E. coli* transformation kit (Zymo Research). Transformants were isolated after overnight growth at 37 °C on LB-Amp plates. Well-separated individual transformants were inoculated in 5.0 mls of LB-Amp media and grown overnight at 37 °C before plasmid isolation with the SV miniprep kit (Promega). Confirmation that LOF phenotypes were plasmid linked was accomplished by transforming the miniprep DNA back into strain HPY120 anew and confirming lack of complementation of the *yvh1*Δ slow-growth phenotype. Candidate plasmids that expressed full-length or near-full-length Yvh1 variants that maintained the slow-growth phenotype were subjected to Sanger sequencing with a primer specific for YVH1 that annealed upstream of the internal *BglII* site in pAB36 to identify any and all changes within the CRD.

### 2.8. Bioorthogonal Noncanonical Amino Acid Tagging (BONCAT) of Nascent Proteins

Cultures of HPY120 transformants were grown in SC -URA media to an A600 of 0.5–1.0. Cells from 150 mL cultures were collected via centrifugation (1990× *g* for 5 min). The cell pellet was washed with 200 mls of sterile dH20 warmed to 30 °C to remove exogenous methionine, and the cells were collected via centrifugation, as described above. The cell pellet was then resuspended in 160 mls of SC -URA Met- media and transferred to a 500 mL flask. The flask was incubated with shaking at 250 rpm for 30 min at 30 °C. At this point, 50 mls of the culture (t = 0) was removed and transferred to a 50 mL conical tube and pelleted via centrifugation (3000 rpm for 5 min in IEC Centra GP8R). The supernatant was discarded, and the pellet was immediately frozen at −80 °C. AHA incorporation of the remaining culture was initiated by the addition of Azido-homoalanine (Click Chemistry Tools (Click, Scottsdale Arizona USA)) to the remaining culture at a final concentration of 20 ug/mL, and the culture was returned to a shaking incubator at 30 °C. Then, 50 mL aliquots were removed at 20 min and 40 min post AHA addition and were processed as described for time 0 samples, and all samples were processed together in parallel.

### 2.9. Isolation of AHA Labeled Lysates

Frozen AHA labeled cultures were thawed on ice and resuspended in 1.0 mL of 1× PBS before being transferred to a 2.0 mL screwcap microfuge tube (Fisher, Hampton, NH, USA). The cells were pelleted via brief centrifugation and then resuspended in 1× PBS containing 1× Halt Protease inhibitor cocktail (Pierce) followed by approximately 25% *v*/*v* 1.0 mm glass beads (Biospec, Bartlesville, OK, USA). The yeast cells were lysed with 4–5 cycles of 45 s full speed in a bead disruptor (Thermo Fastprep FP120 (Biospec Products, Bartlesville Oklahoma USA)) with at least 5 min of cooling on ice between cycles. Whole-cell lysates were obtained via centrifugation at 16,000 rpm at 4 °C for 10 min. The supernatant was removed, carefully so as to not disturb the pellet, and transferred to a clean microfuge tube, and protein concentrations were determined via BCA assays (Pierce) on replicate samples that were then equilibrated to the same protein concentrations with 1× PBS +protease inhibitor as the diluent.

### 2.10. Click Chemistry of AHA Labeled Cell Lysates

THTPA, CuS04 and sodium ascorbate were purchased from Click Chemistry Tools. CW800 alkyne was purchased from LI-COR. Labeling was as described by Click Chemistry tools (https://clickchemistrytools.com/wp-content/uploads/2019/04/Cell-Lysata-Labeling.pdf) (accessed 25 May 2022) with minor modifications. First, 90 µL of 1× PBS was added to 50 µL of the protein concentration-equilibrated AHA labeled yeast lysates. Then, 20 µL of CW800 alkyne (2.5 mM in DMSO) was added, and the sample was mixed via vortexing, followed by the sequential addition of 10 µL of 100 mM THTPA in water, 10 µL of 20 mM CuS04 in water and 10 µL of 300 mM sodium ascorbate in water. The samples were mixed via vortexing after each addition, and the click reaction was allowed to occur for one hour at room temperature in the dark. Then, 600 µL of methanol, 150 µL of chloroform and 600 µL of sterile dH20 were added sequentially followed by mixing via vortexing. The samples were centrifuged at 16,100× *g* for 10 min. The upper aqueous layer was removed and discarded, taking care not to disturb the interface. Next, 450 µL of methanol was added to the interface/organic layer and mixed via vortexing. The proteins were precipitated via centrifugation at 16,100× *g* for 10 min at 4 °C, and the supernatant was discarded. The pellet was washed a second time in 450 µL of 100% methanol followed by centrifugation at 16,100× *g* for 10 min. The supernatant was removed again, and the pellet was allowed to air dry for 10 min in a chemical fume hood. Once dry, the samples were resuspended in a 1× LI-COR sample loading buffer containing 2-mercaptoethanol, before boiling at 95 °C for 5 min in a water bath. Equal volumes of lysate were electrophoresed on 10% SDS-PAGE gel and then transferred to 0.22 um pore nitrocellulose (LI-COR) as recommended. The membrane was briefly submerged in water, and the damp membrane was imaged for 2 min in the CW800 channel on a LI-COR Odyssey to visualize proteins that incorporated AHA clicked to the CW800 alkyne. After CW800 imaging, the membranes were stained with Revert CW700 total protein stain as described by the manufacturer (LI-COR). After sufficient staining, the membrane was re-imaged on both the CW700 and CW800 channels to facilitate merged images. Ratio values for total and newly translated proteins (CW800 counts/CW700 counts) were obtained in Image Studio 4.0, as recommended by the manufacturer. The CW800 and CW700 signals for equal-sized rectangles (excluding the signal for CW800-alkyne free dye) were obtained and standardized to time zero of HPY120 Mrt4GFP/GST at time zero.

### 2.11. Fluorescence Microscopy

HPY120Mrt4GFP cells were transformed with the plasmids indicated, and transformants were grown in SC glucose-URA media overnight. The cells were diluted into the same media and grown at 30 °C for an additional 6 h. The cells were collected via centrifugation at 1350× *g* for 5 min. The supernatant was discarded, and the cells were washed 1× in sterile 1× PBS and repelleted via centrifugation under the same conditions. PBS was discarded, and the cells were resuspended in 2% paraformaldehyde in 1× PBS. The pellet was resuspended via vortexing, and fixation was allowed to occur on an end over end rotator for 15 min at room temperature. The cells were pelleted via centrifugation again, the supernatant was discarded, and the cells were washed 2× in 1× PBS. Then, 5 µL of the washed cells was spotted on the center of a clean microscope Slide (Fisher Cat 22-178-277). At this point, a drop of Fluoroshield mounting medium with DAPI (Abcam) was added to the underside of a glass coverslip. The coverslip was then inverted onto the cells spotted onto the microscope slide and allowed to stand at room temperature for at least 10 min before the coverslip was sealed with clear nail polish. Transformants were imaged on a LeicaDM500C microscope with a 100× oil objective. Fluorescent images were obtained via excitation from a CoolLED PE-300 light source. Images were captured under identical conditions with a Leica K5 sCMOS camera with LasX using the multichannel acquisition module.

### 2.12. Alignment and Topological Description of the Yvh1 CRD

Protein sequences of Yvh1 (NP_012292) and human Dusp12 (NP_009171.1) were subjected to pairwise alignment in T-coffee (http://tcoffee.crg.cat/apps/tcoffee/do:regular) (accessed 25 May 2022). The resulting “Fasta_aln” file was imported into Boxshade (http://www.ch.embnet.org/software/BOX_form.html) (accessed 25 May 2022) Black boxes represent identical residues, and gray boxes represent similarity. The green boxes represent the 7 evolutionarily conserved cysteine residues within the CRD, and the yellow boxes represent the F283 and its corresponding Y268 in human Dusp12. Atomic coordinates of the Yvh1 Cysteine-rich domain were extracted from the Cryo-EM structure of a Rpl10-inserted pre-60S subunit (PDB 6n80) [24]. This accession was entered into the search box at www.rscb.org. On the resulting page, the annotations tab was selected, and Chain D (domain identifier e6n8oY1) was selected and the e6n8oY1 coordinate file was downloaded. This file was then uploaded to the Stride Web interface available online: http://webclu.bio.wzw.tum.de/cgi-bin/stride/stridecgi.py (accessed 25 May 2022) ([25] to produce a visual output which matched what had been previously reported in Figure S6c [24]).

## 3. Results

### 3.1. Yvh1 Evolutionary Conservation and Topology

**Figure 1:**
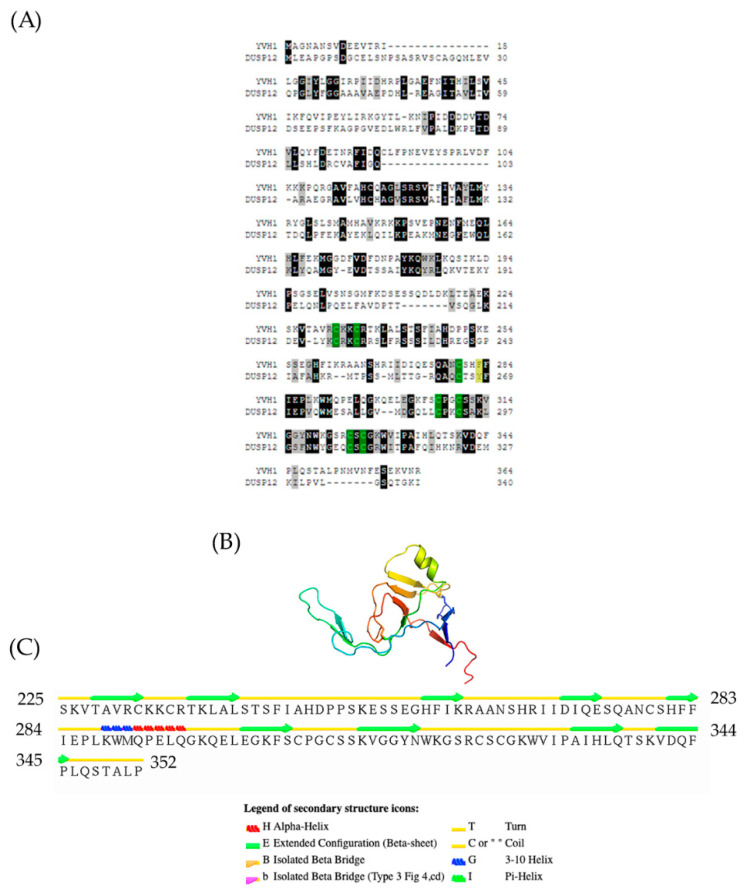
Evolutionary conservation and topology of the Yvh1 cysteine-rich domain. (**A**) Predicted protein sequences of Yvh1 (NP_012292) and human Dusp12 (NP_009171.1) were subjected to pairwise alignment in T-coffee (http://tcoffee.crg.cat/apps/tcoffee/do:regular) (accessed 25 May 2022). The resulting FASTA_.aln file was imported into Boxshade (http://www.ch.embnet.org/software/BOX_form.html) (accessed 25 May 2022). Black boxes represent amino acid, and gray boxes represent similarity. The green boxes represent the 7 evolutionarily conserved cysteine residues within the CRD, and the yellow box represent the F283 and its corresponding Y268 in human Dusp12. (**B**) Atomic structure of the Yvh1 cysteine-rich domain extracted from the Cryo-EM structure of a Rpl10-inserted pre-60S subunit (PDB:6n80). This accession was entered into the search box at www.rscb.org. On the resulting page, the annotations tab was selected, and Chain D (domain identifier e6n8oY1) was selected and the e6n8oY1 coordinate file was downloaded. (**C**) The atomic coordinate file was then uploaded to the Stride Web interface (http://webclu.bio.wzw.tum.de/cgi-bin/stride/stridecgi.py) (accessed 25 May 2022), and Stride was run to produce a visual output of the topological features of the Yvh1 cysteine-rich domain.

An evolutionarily conserved role for Yvh1-dependent Mrt4 removal from pre-60s complexes is well established [4,5]. Results from many laboratories have demonstrated that this ability maps not to the *n*-terminal dual-specificity phosphatase domain (amino acids 1–221, Figure 1A) but to the evolutionarily conserved cysteine-rich domain (amino acids 231–364) at the protein’s C-terminus. The CRD is known to bind to zinc, and prior attempts to mutate the evolutionarily conserved cysteines led to proteins that were unstable in vitro and/or destabilized in vivo [19]. Further demonstrating the extent of conservation and the biological importance of the CRD, the ectopic expression of the human ortholog of Yvh1, Dusp12, was shown to be capable of suppressing the pleiotropic phenotypes of *yvh1Δ* strains as well as Yvh1 alone whether it was the wild-type allele or a variant in which the protein phosphatase activity was inactivated by mutation of the essential catalytic cysteine [19]. How the CRD of Yvh1/DUSP12 promotes Mrt4/MRT04 displacement was unknown until recently. A crystal structure of the human ortholog Dusp12 was deposited (PDB 4JNB) [26], but this structure is limited to the DSP domain and excludes the CRD. Recent reports of high-resolution cryo-EM studies of compositionally defined pre-60S subunits have provided new structural insights into this process. The Yvh1 CRD from pre-60S subunits is composed largely of Beta sheets and turns with a prominent helix from residues 288–295 (Figure 1B,C). Cryo-EM structures of ribosome biogenesis intermediates have proposed a model for how the Yvh1 CRD displaces Mrt4; examining the stalk structure of Mrt4-containing early cytoplasmic intermediate (ECI) and Yvh1-containing later pre-Lsg1 (PL) particles suggests that Yvh1 binding to Mrt4 induces a conformational change that reduces Mtr4’s affinity for rRNA, facilitating its removal [24]. Once Mrt4 is displaced, it allows for the addition of P0 to cytoplasmic pre-60S complexes, as P0 has a higher affinity for 60S rRNA [12]. This model is further supported genetically; variants of Mrt4 with reduced affinity for rRNA (K23 and K69) bypass the slow-growth phenotype associated with Yvh1 deletion, as it is believed that these weaker binders can be spontaneously displaced from pre-60S complexes independently of Yvh1 [4,5].

### 3.2. Expression-Independent Ectopic Suppression of Yvh1Δ Phenotypes

**Figure 2:**
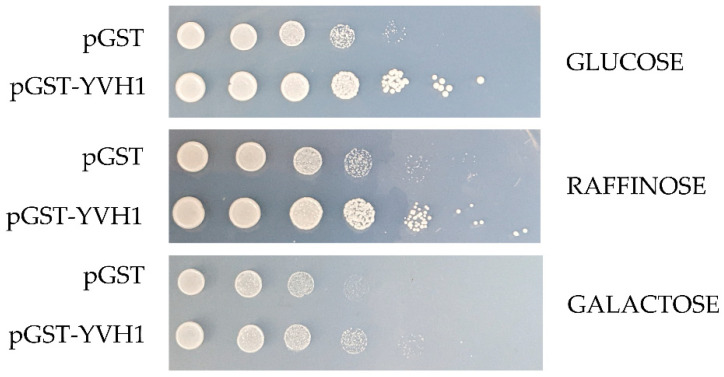
Ectopic expression of GST-YVH1 suppresses the slow-growth phenotype of *yvh1*-deficient strains. Diploid Δ*yvh1/Δyvh1* strains were transformed with the parental pGAL1,10 GST or GST-YVH1 plasmids and selected for uracil prototrophy. Transformants were grown to saturation in Raffinose -URA media, then serially diluted 10-fold before equal volumes were spotted into -URA media containing glucose, raffinose or galactose as the sole carbon source. The transformants were grown at 30 °C for 2 days before being photographed.

The slow-growth phenotype of strains lacking YVH1 is well established [20], and this defect results from defects in ribosome assembly [4,5]. As protein translation is an essential process, we wished to better define the relationship between the expression of a GST-tagged version of YVH1 and the ability to suppress the slow-growth phenotype of yvh1Δ strains. This is of particular relevance when considering other proteins involved in pre-60S maturation. P0 is an essential gene, whereas yeasts lacking RPP1A, RPP1B, RPP2A and RPP2B are viable [27]. In the absence of P1 and P2 proteins, a 30 amino acid C-terminal extension of P0 is proposed to continually interact with translational GTPases and support ribosomal function. If, however, the P0 C-terminal extension is removed in a P1- and P2-deficient background, ribosomes no longer function [13]. Finally, the lethality of galactose-dependent conditional null alleles of P0, as seen in strain D67dGP0 [28], demonstrates that sufficient levels of P0 are required for ribosome biogenesis. We wanted to investigate whether YVH1 expression levels similarly contributed to the ability to suppress the slow-growth phenotype of Yvh1 deficient strains. We previously demonstrated that plasmid-borne GST-tagged variants of YVH1 under the control of a GAL1,10 promoter were sufficient to suppress the slow-growth phenotype under both repressive (glucose as the sole carbon source) and de-repressive (raffinose as the sole carbon source) conditions [14]. We did not ask whether high-level induced expression also suppressed the slow-growth phenotypes. The *yvh1*Δ strain (HPY120) was transformed with the parental GST vector (pEGKG) or a GST-tagged version of YVH1 (identical to pAB36 described in [14]) and grown in liquid cultures to saturation in raffinose-URA media. Transformants were then subjected to a 10-fold dilution series and spotted onto glucose-URA (repressive), raffinose-URA (de-repressive) or galactose-URA (induced) plates and grown at 30 °C for 48 h. Consistent with our previous observations, the expression of GST-YVH1 but not GST alone (Figure 2) was capable of suppressing the slow-growth phenotype under all three conditions. On galactose-containing media, the effect persisted but seemed to be more modest, which we attributed to the overall slower growth of yeast strains with galactose as the sole carbon source. These results suggest that, unlike P0, moderate to high levels of YVH1 are not required to suppress *yvh1Δ* phenotypes and validate the use of low-level expression approaches in subsequent experiments.

### 3.3. Creation of Unbiased Library of Yvh1 Variants within the Cysteine-Rich Domain (CRD)

**Figure 3:**
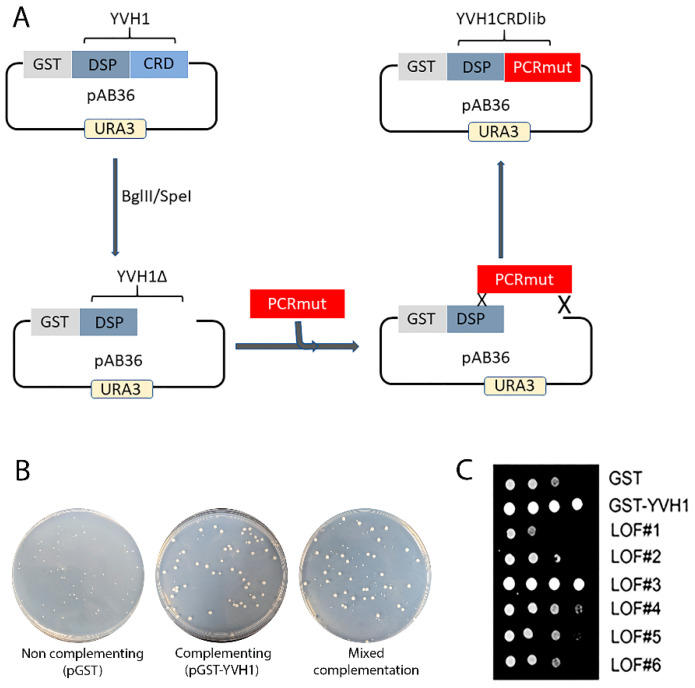
Construction of an unbiased library of YVH1 variants that fail to complement the slow-growth phenotype of strains lacking YVH1. (**A**) Schematic representation of the mutant library construction that restricts amino acid changes to residues 123–364 of Yvh1. The mutated library was generated via Mn-dependent error-prone PCR (EPP) optimized for a suitable mutation rate. The dephosphorylated vector and the CRD EPP library were co-transformed into strain HPY120 (yvh1::HIS3/yvh1::HIS3) to generate gap repair clones via homologous recombination. (**B**) Slow-growing strain HPY120 (yvh1::HIS3/yvh1::HIS3) was transformed by the following plasmids, and the complementation/non-complementation was assessed initially via colony size of the transformants by comparison with the parental vector pGST vector (non-complementing), a GST-YVH1 wild-type variant (complementing) and mixed complementation from the EPP library. (**C**) Loss-of-function (LOF) slow-growing clones from the mixed complementation plates were confirmed via re-streaking on glucose-ura plates followed by serial dilution assays on -URA plates grown at 30 °C. LOF3 initially scored as non-complementing but on further analysis grew comparably to the complementing pGST-YVH1 transformants and was not considered further.

As previous attempts to selectively target evolutionarily conserved residues within the CRD were unfruitful, we initiated an unbiased genetic screen to identify amino acids within the CRD that are essential for Yvh1 function, adopting an error-prone PCR/GAP repair approach [22]. The construction of this mutant library restricted amino acid changes to residues 123–364 of Yvh1; this region was larger than the smallest CRD region sufficient to promote ribosome maturation (aa206–364) [14] but precluded changes within the protein phosphatase domain (Figure 3A). In order to identify individual LOF clones, transformants isolated from the gap repair library (mixed complementation) with growth characteristics that phenocopied the slow growth of HPY120/pGST (non-complementing) transformants (Figure 3B) were isolated by repeated streaking for single colonies on Glucose -Ura plates (complementation under these growth conditions is demonstrated in Figure 2 and in [14]). Slow-growing candidate loss-of-function (LOF) clones were confirmed via spot dilution assays on glucose -Ura plates (Figure 3C). It should be noted that LOF#3 (Figure 3C), which was initially characterized as a slow-growing transformant, was subsequently found to phenocopy GST-YVH1 transformants and represented a false positive in the screen and was abandoned. As we employed a random mutagenesis approach, the EPPCR library was expected to contain several alterations including transitions, transversions and insertions/deletions [29]. As amino acids 206–364 of YVH1 in isolation are sufficient to complement the slow-growth phenotype of *yvh1*Δ strains but a shorter construct (aa291–364) could not [14], we expected that nonsense mutations between residues 206 and 291 would preclude the expression of essential residues and would score as non-informative LOF clones. To enrich for Yvh1 LOF variants that expressed full-length or nearly full-length clones, candidate LOF clones were gown in SC -URA Raffinose media overnight, diluted the next morning into fresh SC -URA Raffinose media and grown for several hours at 30 °C before induction by the addition of galactose to a 4% final concentration. Induction was allowed to proceed for 4–6 h at 30 °C; then, cells were pelleted via centrifugation and whole-cell lysates were obtained by boiling the cell pellets in 1× loading buffer with 2-mercaptoethanol. Whole-cell lysates from candidate LOF clones were compared to whole-cell lysates of transformants expressing GST alone or full-length GST-YVH1 induced similarly by Western blot using anti-GST primary antibodies. Candidate slow-growing LOF variants that expressed a GST-cross reactive band (of any intensity) that comigrated with the band obtained from HPY120/GST-YVH1 were selected for further analysis (Supplemental Figure S1).

### 3.4. Sequencing of Candidate LOF Clones

Plasmids rescued from slow-growing, non-complementing gap repair transformants that produced full-length or near-full-length GST-YVH1 proteins (Supplemental Figure S1) were subjected to DNA sequence analysis with YVH1-specific oligonucleotide primers. A specific LOF clone that contained a single amino acid change (F283L) was selected for further characterization.

### 3.5. YVH1 Affects the Subcellular Localization of Mrt4

**Figure 4:**
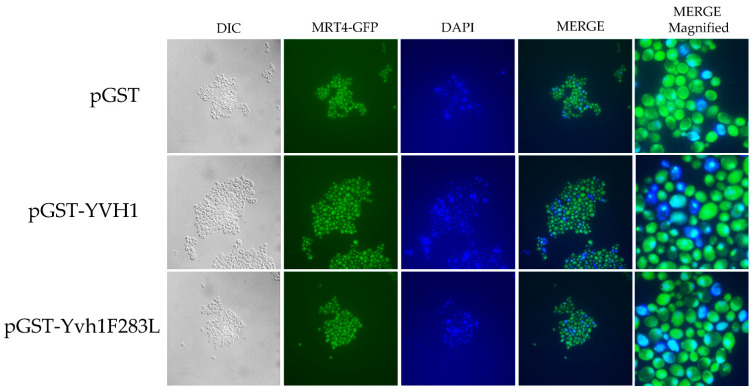
Subcellular localization of Mrt4-GFP in cells expressing YVH1 variants. Strain HPY120MRT4GFP (*yvh1::HIS3/yvh1::HIS3*, Mrt4::GFP) was transformed with the non-complementing pGST, the complementing wild-type pGST-YVH1 or pGST-Yvh1F283L plasmids. Transformants were grown in glucose -URA and then fixed in 2% paraformaldehyde. The cells were then imaged under oil with a 100× objective to acquire differential interference contrast (DIC) microscopy images. The same field was then subjected to fluorescence microscopy for GFP and DAPI channels. Acquisition conditions were identical for all samples, and the merged images were assembled in LasX.

Yvh1 functionally removes cytoplasmic Mrt4 from pre-60S ribosomes, facilitating the addition of the ribosomal stalk [4,5]. Displaced Mrt4 is then efficiently transported back into the nuclear/nucleolar compartment to participate in additional rounds of ribosome maturation [4,5]. We therefore investigated the subcellular localization of Mrt4-GFP in *yvh1*-deficient strains expressing YVH1 alleles episomally as had been done previously. [4,5]. This strain (HPY120 Mrt4-GFP) was transformed with plasmids encoding GST, GST-Yvh1 or GST-Yvh1L283F, and cultures grown in glucose -URA media were imaged microscopically. Transformants containing wild-type GST-YVH1 led to a prominent punctate staining that co-localized with DAPI nuclear staining (Figure 4 middle panel). Slow-growing pGST transformants failed to exhibit punctate Mrt4 GFP signals, and slow-growing pGST-Yvh1F283L transformants also failed to exhibit punctate Mrt4 signals within the nuclear compartment.

### 3.6. pGST-Yvh1F283L Expression Is Reduced but Evident in Yeast

**Figure 5:**
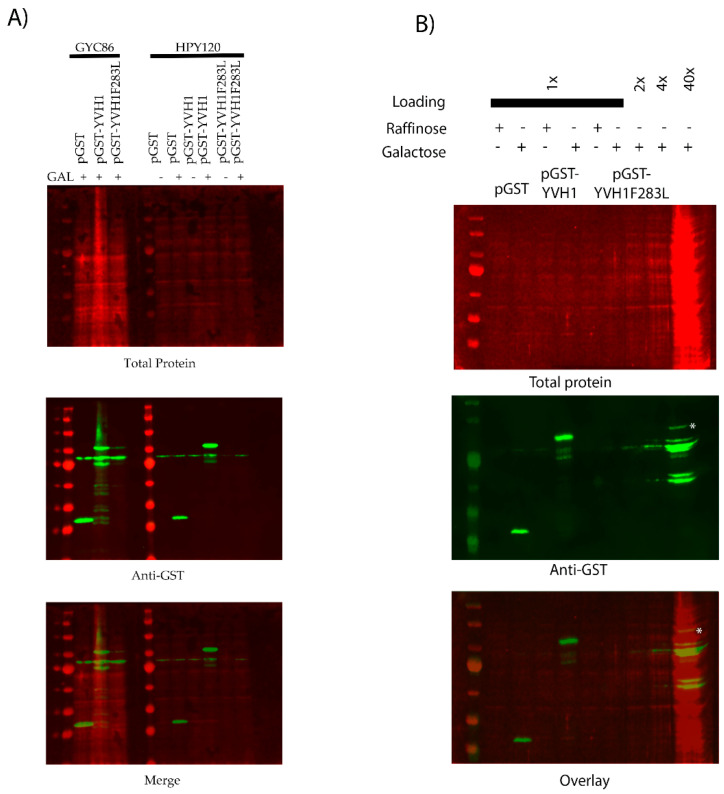
The GST-Yvh1F283L variant is expressed in wild-type and *yvh1*Δ strains. (**A**) Wild-type or *yvh1*Δ strains transformed with the indicated plasmids and equivalent amounts of whole-cell lysates under de-repressive (raffinose) or induced (galactose) conditions were separated on 10% SDS-PAGE Gel and subjected to Western analyses using anti-GST antibodies. Total protein was measured via revert total protein stain (Li-COR). (**B**) Increasing total protein lysates demonstrated that GST-Yvh1F283L is expressed, albeit poorly, in *yvh1*Δ cells. The asterisk denotes a GST-cross reactive band from HP120/pGST-Yvh1F283L that co-migrates with full-length GST-YVH1.

Previous attempts to target specific amino acids within the YVH1 CRD were complicated by protein folding stability issues [19]. In order to argue that Yvh1F283L is incapable of displacing Mrt4 from pre-60s subunits, a demonstration that this mutant protein is in fact stably expressed is required; an alternative explanation is that the F283L allele fails to suppress the slow-growth and cytoplasmic Mrt4 phenotypes as it is not expressed. We should note that in the process of LOF characterization, only clones that expressed full-length or nearly full-length GST-YVH1 variants moved forward (Supplemental Figure S1), suggesting that the YVH1F283L variant was expressed in yvh1Δ strains. To determine the level of expression of GST, GST-YVH1 and GST-YVH1F283L variants, plasmids encoding these alleles were used to transform wild-type or *yvh1*Δ strains. Transformants were grown in liquid cultures overnight under de-repressive conditions (raffinose) and subcultured the next day into the same media for 6 h. A portion of this culture was removed, and galactose was added to the remaining culture, allowing for induction for 4 h. Cell lysates were obtained, and equal amounts of proteins were loaded and subjected to Western analyses with anti-GST antibodies (Figure 5). In wild-type cells, the induced expression of GST-YVH1F283L was observed, albeit at lower levels than GST-Yvh1 or GST alone (Figure 5A compares lanes 4 to 3 and 2). In Yvh1-deficient cells, we failed to detect the expression of GST or GST-YVH1 under de-repressive conditions, but the expression of both variants was evident after galactose induction (Figure 5A, middle panel lanes 7 vs. 8 and lanes 9 vs. 10). Unlike during LOF characterization (Supplemental Figure S1), initially, we did not observe a cross-reactive band under repressive or induced expression for the GST-YVH1F283L variant. In order to resolve this discrepancy, we repeated the Western blot with increasing amounts of whole-cell lysate to see whether increasing total protein levels would allow for the detection of the Yvh1F283L variant. Figure 5B shows a band comigrating with the full-length GST-Yvh1. Collectively, these experiments suggest that the GST-YVH1F283L variant is expressed in both wild-type and *yvh1*Δ strains.

### 3.7. Effects on Nascent Protein Translation

**Figure 6:**
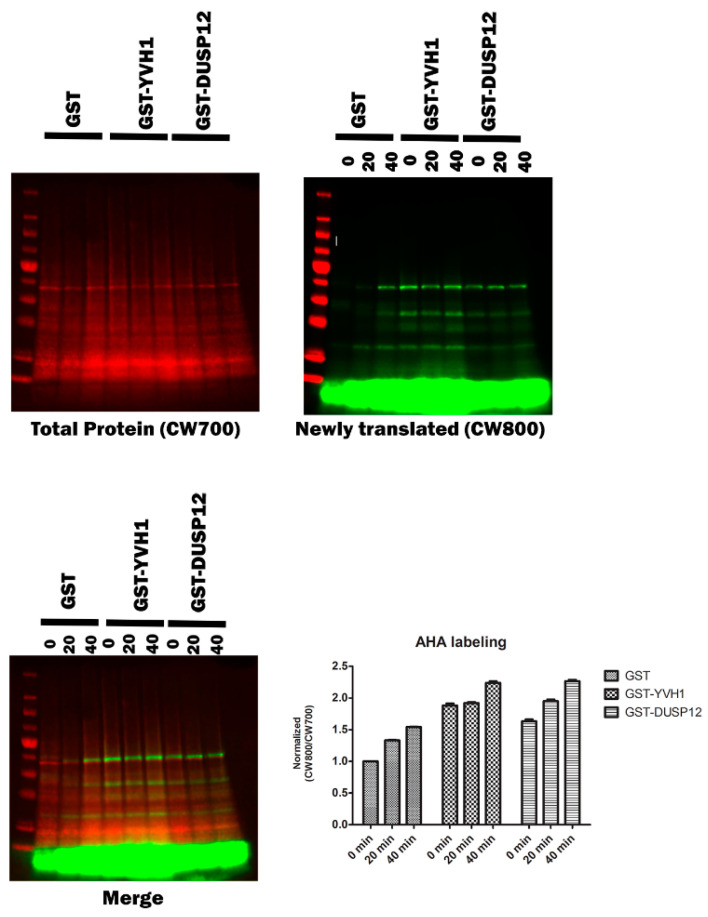
Episomal expression of wild-type Yvh1 or human Dusp12 ortholog rescues nascent protein production defects in cells lacking yvh1. Hpy120MRT4GFP (yvh1::HIS3/yvh1::HIS3, Mrt4::GFP) cells were transformed with empty vector (pGST), fully complementing (pGST-YVH1) or a plasmid harboring the human ortholog of Yvh1, Dusp12. Cells were grown in -Ura media to an A600 of 0.5 to 0.9, pelleted, washed with water and resuspended in -URA Met- media and incubated for an additional 30 min. Azidohomoalanine (a methionine mimetic) was added to the culture at 20 ug/mL at t= 0, and 50 mL aliquots were withdrawn at 0, 20 and 40 min post AHA addition. The cells were collected and frozen. The frozen cells were resuspended in 1× PBS+ protease inhibitors and lysed in a bead beater. Whole-cell lysates containing equivalent amounts of total protein were subjected to copper(I)-catalyzed azide-alkyne cycloaddition (CuAAC) with a CW800 alkyne. Protein samples were resolved on SDS page gel and then transferred to nitrocellulose. Total protein (**upper left panel**) was detected after incubation of the membrane in Revert total Protein stain. Nascent proteins were identified via imaging in the 800 nm (**upper right panel**). The merged image () displays both the total protein and alkyne labeled proteins. Counts from both the CW700 and 800 channels were obtained in Image studio (LI-COR). Quantitation (**lower right panel**) involved taking CW800 (nascent) counts and dividing them by the CW700 counts and setting this ratio to 1 for HPY120/pGST at time zero. Values greater than one are indicative of a relative increase in nascent protein production. Error bars represent SEM for *n* = 3.

Although a role for Yvh1 and Mrt4 in ribosome assembly is well established and defects in ribosomal cytoplasmic maturation are associated with several phenotypes (slow growth and the cytoplasmic retention of Mrt4), these phenotypes are somewhat indirect. To investigate whether Yvh1 loss affects translation directly, we investigated nascent protein production via bioorthogonal non-canonical amino acid tagging (BONCAT) in *yvh1*Δ strains expressing YVH1 variants. Yeast transformants were pre-grown in -URA Met+ media before being transferred to -URA Met- media in the presence of the methionine analog Azahomoalanine (AHA) for varying times. AHA is a methionine mimetic and is efficiently charged to yeast Met tRNAs and incorporated into proteins by the ribosome. AHA incorporation was visualized via click chemistry with a fluorescently labeled alkyne. As demonstrated in (Figure 6), yvh1-deficient transformants expressing GST alone incorporated AHA more slowly than cells expressing GST-YVH1. As the ectopic expression of human Dusp12 has previously been demonstrated to suppress *yvh1*Δ phenotypes (Mrt4 subcellular localization and slow growth), we wanted to determine if this also rescued nascent protein production and observe whether nascent protein production is increased in *yvh1*-defiencent cells ectopically expressing human Dusp12.

### 3.8. The Yvh1F283 Variant Functions as an Expression-Dependent Dominant-Negative

**Figure 7:**
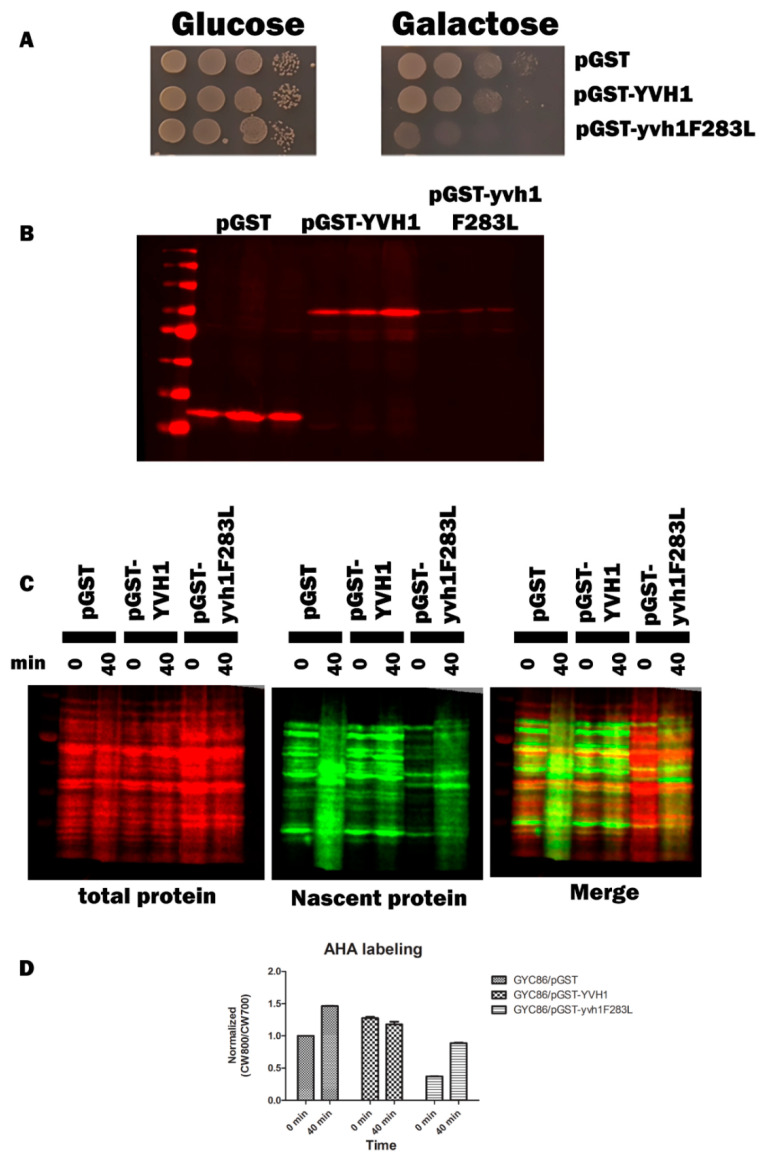
Yvh1F283L functions as an expression-dependent, dominant-negative allele and interferes with protein translation in cells containing endogenous YVH1. (**A**) Wild-type GYC86 was transformed with the plasmids indicated, and three independent transformants for each plasmid were grown to saturation in -URA raffinose media. Ten-fold serial dilutions were made in raffinose -URA media, and equal volumes were spotted onto SC -URA plates containing glucose (1**A left panel**) or galactose (1**A right panel**) as the carbon source. Plates were incubated at 30 °C and photographed 3–4 days later. (**B**) Wild-type GYC86 was transformed with the plasmids indicated and grown in raffinose -URA media to A600 of 0.5 to 1 before induction with 4% galactose for 4–6 h. Cells were collected via centrifugation, and whole-cell lysates were obtained by boiling cell pellets in loading buffer before Western analyses with anti-GST antibodies. (**C**) Nascent protein production in wild-type cells expressing GST, GST-YVH1 or GST-YVH1F283L. GYC86 transformants indicated were grown in URA- Met- media, then transferred to URA- media containing Azahomoalanine (AHA) for the times indicated. Nascent protein production was detected via click chemistry with CW800 alkyne, and total protein was detected using REVERT total protein stain (CW700). (**D**) Quantitation of nascent protein productioninvolved taking CW800 (nascent) counts and dividing it by the CW700 counts and setting this ratio to 1 for HPY120/pGST at time zero. Values greater than one are indicative of a relative increase in nascent protein production. Error bars represent SEM for *n* = 3.

Cryo-EM studies of compositionally defined pre-60S subunits and molecular modeling have identified both the residues of YVH1 that interact with pre-60S complexes and allow for a mechanistic explanation of how Yvh1 displaces Mrt4 from cytoplasmic pre-60S complexes. The CRD of Yvh1 (including residues around F283) form a zinc-binding domain that is wedged between the ribosomal stalk and Tif6 and is centered over the sarcin–ricin loop (SRL) [24], an essential component of the ribosomes that associate with translational GTPases. Comparison of the ribosomal complexes containing Yvh1 and Mrt4 or Mrt4 alone suggested that Yvh1 promotes the displacement of Mrt4 by rearranging the ribosomal stalk, which remodels the interaction between Mrt4 and rRNA, decreasing contacts between the RNA-binding domain, with rRNA loops facilitating its displacement [24]. We then questioned whether the induced expression of GST-Yvh1F283L, which we assume cannot induce the allosteric changes required for Mrt4 displacement, could compete with wild-type YVH1 for binding to pre-60S and in doing so prevent the endogenous YVH1 from displacing Mrt4, precluding the addition of the ribosomal stalk, leading to defects in ribosome assembly. Genetically, we wished to determine whether the induced expression of Yvh1F283L can function as a dominant-negative variant, retaining the ability to associate with pre-60S complexes but lacking the ability to promote the conformational changes required for Mrt4 displacement. Strain GYC86 (YVH1/YVH1) was transformed with galactose-inducible plasmids expressing GST alone, GST-GSTYVH1 or GST-YVH1F283L. Transformants were grown in de-repressing media (Raffinose -URA), and then, equal numbers of transformants were spotted on glucose (to repress the expression of the GST constructs) or galactose media (to induce the expression of the GST construct). Unlike what was seen in Figure 2, the induced expression of GST-Yvh1F283L in cells containing endogenous YVH1 led to a slow-growth phenotype compared to GST alone or GST-Yvh1. (Figure 7A). As was seen previously, the induced expression of GST-Yvh1F283L was reduced compared to GST-Yvh1 or GST alone (Figure 7B). Finally, to confirm that the expression-dependent slow-growth phenotype seen in cells expressing GST-F283L was due to reduced translational efficiency, we monitored nascent protein production in transformants induced to express the transgenes in the wild-type GYC86 background via BONCAT. Figure 7C shows that wild-type transformants induced to express GST showed demonstrated a low basal level of AHA incorporation that increased after 40 min of treatment. The basal expression of transformants expressing wild-type YVH1 was higher than those induced to express GST alone and curiously did not increase after a 40 min treatment. Conversely, the cells induced to express the GST-YVH1F283L variant had a much lower basal translational capability, which increased as a function of time but by 40 min remained below the level basal of cells expressing GST alone. Collectively, these results demonstrate that the YVH1F283L variant is expressed in cells and in the wild-type background can reduce the rate of protein translation, leading to a slow-growth phenotype.

## 4. Discussion

The yeast dual-specificity phosphatase YVH1 gene was initially characterized 30 years ago as a gene whose transcription was induced specifically by nitrogen starvation and whose inactivation led to a severe slow-growth phenotype [20]. Since then, Yvh1’s function has been implicated in a variety of different cellular processes including spore maturation [15], Glycogen accumulation [14], the stabilization of a plasma membrane ATPase [30], the formation of pre-autophagosomal structures after TORC1 inactivation [18] and growth at reduced temperatures [31]. The human ortholog of Yvh1 (DUSP12) has additionally been implicated in binding hsp70 [17] and modulating cellular DNA content [16]. Based on the diversity of cellular processes affected by Yvh1/Dusp12, one might presume that attempts to reconcile all of these phenotypes under a common function would be extremely challenging. A solution to this problem was provided in back to back papers (9,10) that established a role for Yvh1/Dusp12 in cytoplasmic 60S maturation; specifically, Yvh1/Dusp12 are both required to exchange Mrt4/MRTO4 for the ribosomal stalk protein P0/PO prior to the addition of the ribosomal stalk. As translational competency is required for most cellular processes, and life itself, it is not surprising that defects in ribosome assembly would display pleiotropic phenotypes on many cellular pathways.

Yvh1 is clearly a member of the atypical dual-specificity phosphatase family [32]. Surprisingly, the ability to promote ribosome maturation by Mrt4 displacement from pre-60S complexes maps not to the protein phosphatase domain, but to an evolutionarily conserved cysteine-rich domain (CRD) at the protein’s C-terminus. Previous attempts to alter evolutionarily conserved cysteines within the CRD led to proteins that were unstable, precluding the ability to understand how Yvh1 displaces Mrt4 to promote Mrt4/P0 exchange. To the best of our knowledge, despite a well-established role for Yvh1 in cytoplasmic pre-60S maturation, the only non-functional alleles of YVH1 that have been characterized are large deletions within the CRD [4,5,14]. Here, we conducted an unbiased genetic approach attempting to identify critical residues within the Yvh1 CRD that were essential for Yvh1’s biological function. Using error-prone PCR, we generated a full-length or nearly full-length mutant library restricted to a region wholly within the Yvh1 CRD and identified variants that no longer complement the slow-growth phenotype of strains lacking YVH1 (Figure 3). Western analyses of GST-tagged variants allowed us to enrich for full-length or nearly full-length variants by excluding LOF variants resulting from the introduction of nonsense codons or alterations that dramatically affected protein stability (Figure 6, Figure 7 and Figure S1). Sequencing these loss-of-function clones revealed that many clones contained complex mutations, but one specific clone attracted our attention, Yvh1F283L. The fact that this single residue change abolished the ability to complement the null establishes F283 as an important residue for Yvh1’s biological function. Using bioorthogonal non-canonical amino acid tagging, we further demonstrated that Yvh1F283L variants are deficient in nascent protein production, the most direct metric for translational competence.

Although many TAFs were initially identified in genetic screens, more recent advances in Cryo-EM of compositionally defined pre-60S have shed new light on the rearrangements that occur during ribosome maturation. We view these two approaches as highly complementary, but there are still many outstanding questions. Specifically, the Cryo-EM structural model suggests that Mrt4 displacement via YVH1 from pre-60S subunits involves a conformational change in Mrt4 that reduces its affinity for rRNA, which is hard to reconcile with the observation that variants of YVH1 can promote ribosome assembly under transcriptionally repressive conditions where we cannot detect YVH1 immunologically. If the binding of YVH1 to pre-60S complexes releases Mrt4, how is it that repressing the transcription of the YVH1 still allows for the suppression of phenotypes associated with yvh1 loss? A possible explanation is that this results from transcriptional leakage of the of the Gal1,10 promoter. However, in wild-type cells, it is estimated that 200 000 ribosomes are produced per generation [33]. The median number of Yvh1p molecules/cell is much lower (3991), and the median number of molecules of Mrt4p is predicted to be 13,845 under the same conditions [34]. If direct Yvh1p binding to Mrt4p is required to induce a conformational change to help displace MRT4p, it would seem that many 60S complexes would be unable to add the ribosomal stalk. In strains lacking YVH1, how is it that under transcriptionally repressive conditions, the episomal expression of YVH1 under repressive conditions allows for wild-type growth?

Further characterization of Yvh1F283L established it as an expression-dependent, dominant-negative allele. It is important to note, however, that F283L was initially identified as a loss-of-function variant that failed to suppress phenotypes associated with YVH1 loss. It is certainly possible that additional variants targeting F283 or residues proximal to F283 may function as even better dominant-negatives, and we are currently evaluating this possibility experimentally. Nevertheless, we believe that any dominant-negative allele has utility in furthering our understanding of ribosome assembly. Although ribosome assembly is often presented as a linear obligate series of rearrangements to generate structurally identical translationally competent ribosomes, there is increasing evidence for ribosomal heterogeneity [35]. Biochemically demonstrating alterations in the stoichiometry of very large molecular complexes is experimentally challenging but can be accomplished genetically by the expression of factors that interfere with normal ribosome biogenesis. As ribosome biogenesis is evolutionarily conserved, the likelihood that modulators of ribosome assembly identified in yeast will function in other eukaryotic cells is quite good. For example, the siRNA depletion of Dusp12 in HeLa cells leads to the increased cytoplasmic accumulation of MRT04 [5]. Why would one want to investigate the biological consequences of induced ribosomal heterogeneity? We believe that this is of particular interest as there is preliminary evidence that compositionally heterogenous ribosomes have differential abilities to translate specific proteins in yeast [36] and in human cells (reviewed in [37]). Furthermore, the remodeling of ribosomal composition may occur in response to changing environmental conditions [38] or during embryonic development. The ability to evaluate the biological consequences of alterations in ribosome assembly may also have direct relevance to human health. Ribosomopathies are a group of congenital human disorders most commonly caused by haploinsufficiency or caused by defects in ribosome biogenesis [39] that predispose patients to cancer. As the loss of YVH1 is not lethal in yeast and precludes the addition of the ribosomal stalk, the selective loss or inactivation of YVH1/Dusp12 function may allow for better understanding of the consequences of compositionally distinct ribosomes. This is of particular importance, as all steps of protein synthesis are susceptible to dysregulation during cancer development [40], and the overexpression of DUSP12 (the human ortholog of YVH1) has been identified as a candidate oncogene in human liposarcomas [41].

## 5. Conclusions

Ribosome assembly is an evolutionarily conserved process that contributes to all biological processes through the production of cellular proteins. When dysregulated, this process can have dramatic effects and can lead to several human malignancies. Previous genetic approaches and more recent Cryo-EM studies have begun to elucidate the process of ribosome maturation.

Here, we focused on a protein that functions late in the process of 60S cytoplasmic maturation, YVH1. Previous genetic studies implicated Yvh1p as essential to the removal of the Mrt4 trans-acting factor. Using an unbiased genetic approach, we isolated a previously uncharacterized variant of YVH1 that fails to displace Mrt4, preventing the addition of the ribosomal stalk. We additionally demonstrated that this variant can function as a dominant-negative by perturbing endogenous YVH1 function and envisioned that this variant and variants developed from it could serve as novel probes to selectively inhibit a step of 60S maturation in yeast and in other eukaryotes.

## Data Availability

Not applicable.

## Supplementary Materials

The following supporting information can be downloaded at: https://www.mdpi.com/article/10.3390/biology11081246/s1, **Figure S1.** *Representative Selection for full-length or near full-length LOF clones.* Candidate slow-growing loss of function clones were grown in raffinose-URA media, then induced by the addition of galactose to 2%. Cell pellets were obtained, resuspended in 1X loading buffer and subjected to Western analyses with anti-GST antibodies. Clones that expressed proteins that co-migrated with the full-full length GST-YVH1 (lane 3) are identified by asterisks and were considered for further analyses. Using the A6 anti-GST antibody (Santa Cruz) we always observed a non- specific band that migrated slightly faster than the full length GST-YVH1 and we often used this band as an internal standard to evaluate relative expression. For example, lanes 7 and 9 express lower amounts of the GST-YVH1 constructs, but using the non specific band it appears that these lanes are underloaded for total protein.
