# Supplementary material for: Mutational Analyses of the Cysteine-Rich Domain of Yvh1, a Protein Required for Translational Competency in Yeast
Source: Biology (Basel). 2022 Aug 22;11(8):1246. doi: 10.3390/biology11081246 (PMC9404827; doi:10.3390/biology11081246)
Supplement: Supplementary file 1 [file biology-11-01246-s001.zip › biology-1766903-supplementary.pdf]

biology

Supplemental Figures

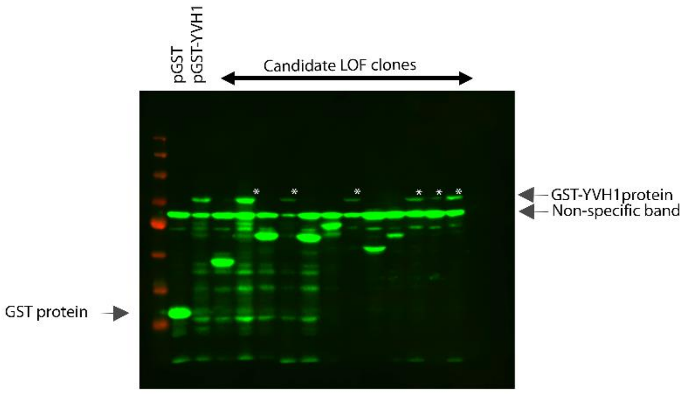

Supplemental Figure S1. *Representative Selection for full-length or near full-length LOF clones.* Candidate slow-growing loss of function clones were grown in raffinose-URA media, then induced by the addition of galactose to 2%. Cell pellets were obtained, resuspended in 1X loading buffer and subjected to Western analyses with anti-GST antibodies. Clones that expressed proteins that co-migrated with the full-full length GST-YVH1 (lane 3) are identified by asterisks and were considered for further analyses. Using the A6 anti-GST antibody (Santa Cruz) we always observed a non- specific band that migrated slightly faster than the full length GST-YVH1 and we often used this band as an internal standard to evaluate relative expression. For example, lanes 7 and 9 express lower amounts of the GST-YVH1 constructs, but using the non specific band it appears that these lanes are underloaded for total protein.
